# Facile, Scalable, Eco-Friendly Fabrication of High-Performance Flexible All-Solid-State Supercapacitors

**DOI:** 10.3390/polym10111247

**Published:** 2018-11-11

**Authors:** Jincy Parayangattil Jyothibasu, Rong-Ho Lee

**Affiliations:** 1 Department of Environmental Engineering, National Chung Hsing University, Taichung 402, Taiwan; jincycusat@gmail.com; 2 Department of Chemical Engineering, National Chung Hsing University, Taichung 402, Taiwan

**Keywords:** kapok fiber, polypyrrole, carbon nanotube, freestanding electrode, all-solid-state symmetric supercapacitor

## Abstract

A highly porous freestanding supercapacitor electrode has been fabricated through a simple, inexpensive, bulk-scalable, and environmentally friendly method, without using any extra current collector, binder, or conducting additive. Benefiting from its unique micro-tubular hollow structure with a thin cell wall and large lumen, kapok fiber (KF) was used herein as a low-cost template for the successive growth of polypyrrole (PPy) through in situ chemical polymerization. This PPy-coated KF (KF@PPy) was blended with functionalized carbon nanotubes (*f*-CNTs) to form freestanding conductive films (KF@PPy/*f*-CNT) through a simple dispersion and filtration method. The hybrid film featuring the optimal composition exhibited an outstanding areal capacitance of 1289 mF cm^−2^ at a scan rate of 5 mV s^−1^. Moreover, an assembled all-solid-state symmetric supercapacitor featuring a PVA/H_2_SO_4_ gel electrolyte exhibited not only areal capacitances as high as 258 mF cm^−2^ (at a scan rate of 5 mV s^−1^) but also excellent cycling stability (97.4% of the initial capacitance after 2500 cycles). Therefore, this efficient, low-cost, scalable green synthesis strategy appears to be a facile and sustainable way of fabricating high-performance flexible supercapacitors incorporating a renewable cellulose material.

## 1. Introduction

The increasing demand for portable and flexible electronic devices in modern society is triggering the development of lightweight, ultrathin, flexible, inexpensive, and sustainable energy storage systems that operate with high performance [1,2,3]. Among such energy storage systems, supercapacitors (SCs) have attracted a great deal of attention because of their high power density, long cycle life stability, fast charge/discharge rates, and good operational safety [4,5]. The fabrication of conventional SC electrodes generally involves inactive components (e.g., a metallic current collector, a polymeric binder, and conducting additives) that do not contribute to the charge storage; instead, they can make the whole device too heavy and bulky to meet the practical requirements for portable devices. Moreover, the liquid electrolytes used in conventional devices pose serious leakage problems and require the use of complex encapsulation processes, further increasing the weight and cost of the devices [3,6,7,8]. All-solid-state SCs, which employ gel polymer electrolytes instead of aqueous electrolytes, provide more design opportunities for the fabrication of thinner and lighter devices, because they do not require as rigid packaging as their liquid counterparts [9,10]. Thus, there is a challenge to develop inexpensive, scalable, and environmentally friendly strategies for the fabrication of lightweight and flexible all-solid state SC devices with high energy/power densities, such that they would be compatible with portable and miniaturized energy storage systems [11,12,13,14]. Because the electrode plays a key role in determining the form factors and electrochemical properties of all-solid-state SCs, a suitable architecture for the design of the electrode might ensure the fabrication of useful all-solid-state SCs. Ideally, readily available, inexpensive, and sustainable materials would be used as substrates when fabricating freestanding and binder free electrodes, thereby providing affordable and eco-friendly all-solid-state SCs.

In recent years, increased scientific interest has turned toward the use of cellulose fiber (CF)—an abundant, renewable, recyclable, and biodegradable material—as a substrate for the preparation of freestanding electrodes [15,16,17,18,19]. An additional attraction of CF is that its large number of surface functional groups enables the fibers to bind with electrochemically active materials, e.g., carbon nanotubes (CNTs) [20,21], graphene/graphite [5,13,22,23,24], conducting polymers [16,17,20,22,25,26], and metal ions and oxides [18,27,28] to form freestanding electrodes. Cellulose-based freestanding electrodes provide many desirable features to all-solid-state SCs, including high electrochemical performance, mechanical strength, light weight, flexibility, bulk scalability, and biodegradability. Various cellulose-based substrates have been investigated for their application in SC electrodes, including commercial cellulose paper [20,27], cellulose pulp [22], cotton textiles [21,29,30,31], cellulose nanocrystals [16,26], cellulose nanofibers [19,32], and bacterial cellulose [7,17,18,23,28]. Cellulose-based conductive composites, with their highly porous continuous three-dimensional (3-D) network structures, can provide clear pathways for rapid electron and ion transport and, thereby, achieve excellent electrochemical performance [23,33]. Even though cellulose itself is an environmentally friendly green material, the extraction of cellulose for the production of various cellulose-based materials (e.g., cellulose paper, nano cellulose, cellulose pulp, and cellulose nanocrystals) often involves toxic chemicals and intensive possessing conditions, resulting in the generation of huge amounts of waste materials that are harmful to the environment and human health [34,35,36]. Hirotaka and co-workers proposed an eco-friendly method for the fabrication of more affordable paper composites, using recycled waste pulp from newspapers as a substitute for virgin pulps [5]. Nevertheless, the electrochemical data they furnished for the reduced graphene oxide (rGO)/cellulose paper composite prepared by embedding rGO in recycled waste pulp were insufficient to make any assessments of practical utility. Moreover, it is likely that the ink and other inactive additives in the waste paper pulp might seriously impair the performance of the electrode materials and, therefore, they must be eliminated completely prior to use. Bacterial nanocellulose (BNC) has been chosen as an alternative to plant-derived cellulose in many studies of the preparation of SC electrodes [17,18,23,28,37]. Despite its superior properties compared to regular cellulose papers (e.g., biosynthetic origin; high purity and crystallinity), the current price of BNC is too high to make it commercially attractive and viable for bulk production [38]. Moreover, the production of BNC represents a step back from the main principles of green chemistry [36]. The preparation of cotton fabric–based SCs has been a straightforward, simple, scalable, and eco-friendly approach for the fabrication of green energy storage systems [21,30,31]. Nevertheless, the areal capacitances of these SCs remain too low, because of their low active material loading, for practical applications in SCs. For example, the area specific capacitance reported for an all-solid-state SC fabricated using PPy/CNT/cotton fabric was as low as 50.09 mF cm^−2^ at 25 mV s^−1^ [39]. Thus, the search continues for an alternative, cheap, and readily available cellulose substrate, coupled with a simple and less energy intensive fabrication process, for the mass production of inexpensive green energy storage devices. For example, natural transverse section slices of wood have been introduced as new flexible porous substrates for inexpensive SCs; such a PPy-coated wood transverse section slice composite electrode has exhibited an areal capacitance of 0.61 F cm^−2^ and retained 87.5% capacitance after 5000 cycles [34]. Nevertheless, the quest for inexpensive and sustainable raw materials for the development of more affordable and environmentally friendly high-performance energy storage systems remains a challenge.

Kapok fiber (KF) is a single-celled natural CF featuring a significantly homogeneous hollow microtubular structure [40,41]. It has been used as a natural bio-template for the preparation of microtubular composites because it has a large specific surface area and good surface activity, with abundant active hydroxyl (OH) groups to bind with various nanomaterials (e.g., conducting polymers, metal oxides) [42,43,44]. When pseudocapacitive materials have previously been coated on KF and used as electrode materials for SCs, superior electrochemical properties were obtained because of the unique structure of the KF [40]. Nevertheless, high-temperature carbonization of the KF has often been required to further enhance the electrochemical properties [40,43]. Moreover, these previous studies conducted using KF composites employed a traditional electrode fabrication process, with the composite material mixed with binders and conducting additives and then cast on metallic current collectors to evaluate the electrochemical performances in aqueous electrolytes. This cumbersome electrode fabrication process and the use of an aqueous electrolyte are major drawbacks for the practical applications of such materials in portable and flexible electronics. KF is one of the lightest natural CFs in the world, with a density of 0.3 g cm^−3^, because of its unique hollow structure with a hollow ratio as high as 97% [41]. Its density is very low compared with other natural CFs—for example, cotton (1.5–1.6 g cm^−3^) [45] and bacterial cellulose (1.25 g cm^−3^) [46]. Taking into account this low density, high porosity, and large specific surface area, it is reasonable to assume that KF composites could be employed to fabricate freestanding electrodes for sustainable, lightweight, inexpensive all-solid-state SCs. To the best of our knowledge, however, no research has been conducted previously to take advantage of the unique properties of KF to fabricate freestanding electrodes for all-solid-state flexible SC devices.

Among the various conducting polymers, PPy is a promising material for high-performance SC applications, because of its high conductivity, good redox properties, ease of synthesis, low price, environmental stability, and nontoxicity [10,23,30]. Nevertheless, the volumetric change during its charge/discharge processes caused by ion doping and de-doping, may lead to structural damage, resulting in poor cycle stability [28,37,47]. Moreover, the dense structure of a PPy film impedes ion diffusion into the inner active material, leading to decreased power capability and large scale capacitance degradation at high charge/discharge rates. Combining PPy with highly conductive CNTs to form hybrid electrodes can be an effective strategy for enhancing the electrical conductivity, specific capacitance, rate capability, and cycling stability [30,39,48,49,50]. CNTs, with their extensive porous network structure and high conductivity, can accelerate the rates of electron transport and electrolyte ion diffusion, thereby improving the rate capability and cycle life of the electrode. Furthermore, CNTs can also serve as structural reinforcing materials to enhance the mechanical strength of PPy-CNT composites, thereby eliminating the need for current collectors, binders, and conductive additives when fabricating the electrodes. 

In our study, kapok fiber (KF), having a unique micro-tubular hollow structure, was used as a low-cost template for the successive growth of polypyrrole (PPy). KF provides two surfaces—internal and external—for the polymerization of PPy; in contrast, in the case of commonly used carbon fiber–based flexible substrates, PPy can polymerize only on the outer surface, thereby limiting the PPy loading [51,52,53,54,55,56,57]. Furthermore, KF have has an average density of only approximately 0.3 g cm^−3^ [41], making it about seven times lighter than dry carbon fibers (1.7–2.0 g cm^−3^) [58] and, thus, more suitable for preparing light-weight freestanding electrodes. The PPy-coated KF (KF@PPy) was blended with functionalized carbon nanotubes (*f*-CNTs) and the freestanding hybrid film was fabricated through simple dispersion and vacuum filtration. The resulting hybrid film was highly porous, with good mechanical properties that maintained the desired shape and thickness. The thickness of the film can be readily controlled by simply varying the concentration of the KF@PPy and *f*-CNTs mixture or the filtration volume. This approach provided a high mass loading of the active materials per unit area. This advantage of enhancing the areal mass loading by controlling the thickness is not possible for carbon fiber paper or carbon cloth of a fixed thickness. These KF@PPy/*f*-CNT hybrid films, when used directly as freestanding electrodes, exhibited excellent electrochemical properties. To test the practicality of these hybrid films in all-solid-state SCs, a symmetric SC device was assembled by sandwiching a poly(vinyl alcohol) (PVA)/H_2_SO_4_ electrolyte between two identical pieces of KF@PPy/*f*-CNT hybrid film. The device exhibited an outstanding areal capacitance (258 mF cm^−2^ at a scan rate of 5 mV s^−1^) and excellent cycling stability (97.4% capacitance retention after 2500 cycles at 25 mA cm^−2^). Therefore, this efficient, inexpensive, and scalable green synthesis strategy assists the development of sustainable and affordable SCs.

## 2. Materials and Methods 

### 2.1. Chemicals 

CNTs were obtained from C-nano Technology (Beijing, China). KF (Ceiba pentandra) was purchased from Uni-onward (New Taipei City, Taiwan). Pyrrole (Alfa Aesar, 98%, Heysham, Lancashire, UK) was distilled under reduced pressure and stored at low temperature under a N_2_ atmosphere prior to use. Ferric chloride hexahydrate (FeCl_3_**·**6H_2_O) purchased from Sigma–Aldrich (St. Louis, MO, USA). Sodium chlorite (NaClO_2_) and nitric acid (HNO_3_) were procured from Showa Chemical Industry (Tokyo, Japan). Acetic acid (CH_3_COOH), sulfuric acid (H_2_SO_4_), and PVA were purchased from Sigma–Aldrich (St. Louis, MO, USA) and used as received. High-purity deionized water was used in all experimental processes.

### 2.2. KF/Polypyrrole Composites

Raw KF was pretreated with NaClO_2_ solution to remove the waxy coating and create a hydrophilic surface, as described previously [59]. This pretreated KF was used in all of the following experiments. Pretreated KF (200 mg) was dispersed in deionized water (150 mL) and then pyrrole (1.0 mL) was added. The mixture was stirred for 30 min in an ice bath. Aqueous FeCl_3_**·**6H_2_O (50 mL) was added dropwise under vigorous stirring to initiate the polymerization of pyrrole. The polymerization was performed for 6 h; the molar ratio of pyrrole to FeCl_3_**·**6H_2_O was 1:1.5. The resulting black composite was washed several times with alcohol and deionized water to remove the byproducts and excess reagents. It was then collected through vacuum filtration and dried under vacuum at 60 °C for 24 h. This sample is named herein as KF@PPy. The amount of PPy chemically deposited on the KF surface was determined by weighing the KF before and after the PPy polymerization reaction under standard conditions. The PPy weight percentage (*W*_PPy_%) was calculated as follows (Equation (1)):

(1) $$W_{PPy\;}\%=\left(\frac{W_{f}-W_{i}}{W_{f}}\;\right)\;\times \;100\%$$

where *W*_i_ and *W*_f_ are the initial and final weights of the KF before and after PPy polymerization, respectively.

### 2.3. KF@PPy/f-CNT Freestanding Films

Carboxylated CNTs (*f*-CNT) were prepared by heating the CNTs under reflux in 3 M HNO_3_ for 14 h at 90 °C. The sample was then sonicated in an ultrasonication bath for 1 h. The resulting modified CNTs were filtered through a cellulose acetate membrane and washed thoroughly with deionized water to neutral pH, then dried under vacuum at 100 °C for 24 h. The KF@PPy and *f*-CNT samples were dispersed separately in desired amounts of deionized water and subjected to ultrasonication to form homogeneous dispersions. These homogeneous dispersions were combined, with vigorous stirring for 30 min and sonication for 5 min to ensure sufficient mixing. The as-obtained suspension was maintained at rest for 2 h to ensure that the *f*-CNTs assembled with the KF@PPy composite. The suspension was vacuum filtered to form a filter cake, which was washed several times with deionized water. The washed filter cake was dried (60 °C, 12 h) and peeled off to give a freestanding membrane. To determine the ratio of KF@PPy and *f*-CNT the provided the best electrode performance, KF@PPy/*f*-CNT freestanding membranes were prepared at weight ratios of 1:2, 1:1, and 2:1; they are named KF@PPy/*f*-CNT12, KF@PPy/*f*-CNT11, and KF@PPy/*f*-CNT21, respectively.

### 2.4. Characterization

Fourier transform infrared (FTIR) spectra of the samples were recorded using a Nicolet Avatar 320 spectrometer (Nicolet, Madison, WI, USA), armed with an attenuated total reflectance (ATR) sampling tool, scanned over the wavenumber range from 650 to 4000 cm^−1^. Thermogravimetric analysis (TGA, PerkinElmer Pyris1, Shelton, CT, USA) was performed under a N_2_ atmosphere at heating rate of 10 °C min^−1^. X-ray diffraction (XRD) analysis was performed using a Rigaku diffractometer (Rigaku RINT 2000, Tokyo, Japan) with Ni-filtered Cu Ka radiation. The morphologies and microstructures of composites were observed using scanning electron microscopy (SEM, JSM 7401F; operating voltage: 3 kV, JEOL, Tokyo, Japan). Prior to analysis, the samples were mounted on the microscope stubs using double-sided conductive tape and coated with a thin layer of evaporated gold.

### 2.5. Electrochemical Measurements

The electrochemical properties of the freestanding electrodes were tested using cyclic voltammetry (CV), galvanostatic charge/discharge (GCD), and electrochemical impedance spectroscopy (EIS) techniques on a CHI6273D electrochemical work station (CH Instruments, Inc., Austin, TX, USA). A three-electrode system was used to evaluate the performances of the as-prepared composites in aqueous 1 M H_2_SO_4_. A piece of the freestanding film (active area: 1 cm^2^) was used directly as the working electrode. A saturated calomel electrode (SCE) and platinum foil were used as the reference and counter electrodes, respectively. CV was performed in the voltage range from −0.2 to +0.8 V at scan rates of 5, 10, 20, 30, 40, and 50 mV s^−1^. EIS was performed at the open-circuit potential with an amplitude of 5 mV over a frequency range from 0.01 Hz to 100 kHz. The GCD experiments were performed in the potential window from −0.2 to +0.8 V with applied current densities of 2, 3, 4, 5, 6, and 8 mA cm^−2^. Areal capacitance of the freestanding electrodes were calculated from their CV data, according to the Equation (2):

(2) $$C_{A}=\;\frac{S}{2\nu \;\times \Delta \;U\;\times A_{S}\;}$$

where *C*_A_ (F/cm^2^) is the areal capacitance, *S* is the area enclosed by the CV curve within the potential window, *ν* is the scan rate (mV s^−1^), ∆*U* is the potential window (V), *A*_s_ (cm^2^) is the area of single electrode.

Alternatively, the areal capacitances of the freestanding electrodes were calculated from the charge/discharge curves, using the Equation (3):

(3) $$C_{A}=\frac{I\;\Delta t}{A_{S}\;\Delta V}$$

where *I* is the discharge current (A), Δ*t* is the discharge time (s), *A_s_* is the area of the freestanding electrode (cm^2^), and Δ*V* is the potential window (V).

Volumetric capacitance *C*_v_ (F/cm^3^) calculated as follows Equation (4):

(4) $$C_{v}=\;\frac{C_{A}}{d\;}$$

where d is the thickness of the single electrode.

### 2.6. Fabrication of All-Solid-State SC

A H_2_SO_4_/PVA gel electrolyte was prepared by mixing PVA powder (1 g), H_2_SO_4_ (1 g), and deionized water (10 mL) at 85 °C under stirring to form a transparent and homogenous solution. Two KF@PPy/*f*-CNT films (each with an area of 1 × 1.5 cm) as electrodes were immersed into the H_2_SO_4_/PVA solution for 10 min. After solidification of the gel electrolyte, the two electrodes were pressed together, face to face, to form an all-solid-state flexible symmetric SC. The assembled device had a thickness of approximately 0.075 cm. The electrochemical performance of the all-solid-state symmetric device was evaluated using a CHI6273D electrochemical work station. The cell (device) areal/volumetric capacitance (*C*_cell_) was calculated from the CV data, according to the Equation (5):

(5) $$\;C_{\mathrm{cell}}=\;\frac{S}{2\nu \;\times \Delta \;U\;\times A_{t}}$$

where *C*_cell_ (F cm^−2^) is the areal capacitance, *S* is the area enclosed by the CV curve within the potential window, ν is the scan rate (mV s^−1^), ∆*U* is the potential window (V), *A*_t_ (cm^2^) is the total area of both electrodes in cell.

The areal capacitance (*C*_cell_, F cm^−2^), energy density (*E*, mW h cm^−2^), and power density (*P*, mW cm^−2^) of the assembled SC device were calculated from the charge/discharge curve, according to the Equations (6), (7) and (8):

(6) $$C_{\mathrm{cell}}=\frac{I\;\Delta t}{A_{t}\;\Delta V}$$

(7) $$E=\frac{1}{2}\frac{C_{\mathrm{cell}}V^{2}}{3.6}$$

(8) $$P=\frac{E\;\times 3600}{\Delta t}$$

where *I* is the discharge current (A), Δ*t* is the discharge time (s), *A*_t_ is the total area of both electrodes in cell (cm^2^), and Δ*V* is the potential window (V).

Volumetric capacitance *C*_v-cell_ (F/cm^3^) calculated as follows Equation (9):

(9) $$C_{v-\mathrm{cell}}=\;\frac{C_{\mathrm{cell}}}{d\;}$$

where d is the thickness of the all-solid-state symmetric supercapacitor cell.

## 3. Results and Discussion

**Scheme 1:**
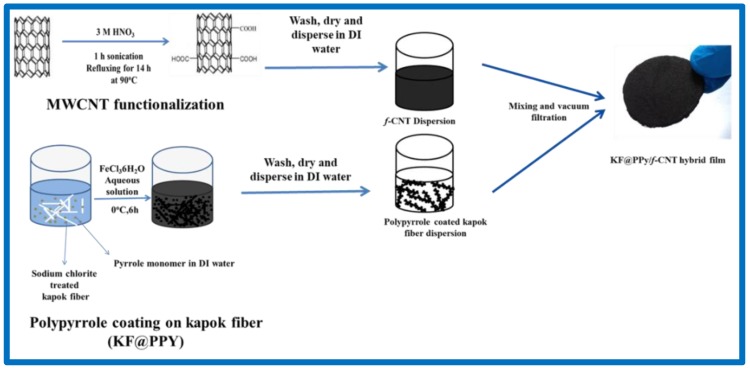
Schematic representation of the fabrication of KF@PPy/*f*-CNT freestanding hybrid films.

Scheme 1 presents a schematic representation of the fabrication of the KF@PPy/*f*-CNT hybrid films. The raw KF was hydrophobic because of the presence of a thick layer of wax on its surface. Thus, pretreatment with sodium chlorite was performed to remove the wax layer and induce hydrophilicity. This pretreated KF was used throughout these experiments. To coat PPy onto the KF, pyrrole was added to a dispersion of KF and then polymerization was initiated by adding FeCl_3_**·**6H_2_O solution. The color of the KF turned from white into black, indicating the successful incorporation of PPy on the KF surface. This PPy-coated KF (KF@PPy) was blended with *f*-CNTs, through simple dispersion and vacuum filtration, to form freestanding conducting films (KF@PPy/*f*-CNT). Raw CNTs exist in bundles and have poor dispersibility in water; they need to be oxidized to form good dispersions. The oxidization is usually performed under harsh conditions, involving intensive ultrasonic treatment in a mixture of the strong acids HNO_3_ and H_2_SO_4_. Such treatment is not only hazardous to the environment but also severely damages the electronic and mechanical properties of the CNTs [60,61]; it can also result in severe fragmentation of the CNTs into shorter tubes, which are less likely to form entangled porous networks in the freestanding films [50,62]. Therefore, in this study we functionalized CNTs under relatively mild conditions: heating the raw CNTs under reflux in 3 M HNO_3_ at 90 °C for 14 h. CNT functionalization in this manner generally causes less damage to the tubes, allowing them to retain their original electronic and mechanical properties. These *f*-CNTs, featuring abundant oxygen-containing functional groups, had good dispersibility in water. In addition, we expected these functional groups to enhance the interaction between the *f*-CNTs and PPy [60,63], thereby potentially forming a highly interconnected porous network structure after simple vacuum filtration. The amount of PPy deposited on the KF was calculated to be approximately 68 wt %. Based on this value, the PPy loading per unit area of the KF@PPy/*f*-CNT11 freestanding electrode was estimated to be 3.6 mg cm^−2^. The PPy loading achieved for our electrode is much higher than those achieved for many previously reported PPy-based freestanding electrodes [8,25,51,52,53,54,55,56,57,58,64,65,66,67]. The mass fraction of KF was low, approximately 16 wt %, in the freestanding electrode.

**Figure 1:**
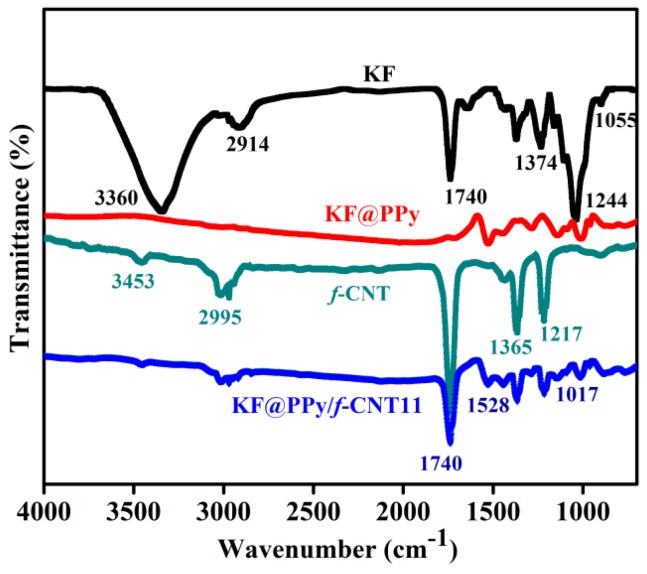
FTIR spectra of KF, KF@PPy, *f*-CNT, and KF@PPy/*f*-CNT11.

Figure 1 displays FTIR spectra of the KF, KF@PPy, *f*-CNTs, and KF@PPy/*f*-CNT11 composites. The spectrum of the pretreated KF exhibited three characteristic absorption peaks for acetyl ester groups: at 1740 cm^−1^ [stretching of ester carbonyl (C=O) group], 1374 cm^−1^ (C–H bending in –O(C=O)–CH_3_), and 1244 cm^−1^ (C–O stretching in ester) [3,42]. The intense bands near 1055 cm^–1^ corresponded to skeletal vibrations of the C–O–C functional groups of the pyranose rings [18,28]. The absorption peak at 3360 cm^–1^ and a broad band at 2914 cm^–1^ can be ascribed to the stretching vibrations of the OH groups and the asymmetric stretching vibration of C–H units, respectively, in the cellulose of KF [68]. The FTIR spectrum of KF@PPy presented primarily the bands typical of PPy. The absorption peaks at 1528 and 1436 cm^−1^ can be attributed to C=C and C–N stretching vibrations, respectively, of the pyrrole rings [8,10,25,69]. The peaks near 1292 cm^−1^ represent =C–N stretching vibrations of the pyrrole rings. The peak at 1149 cm^−1^ corresponds to the breathing vibration of the pyrrole rings. The peak associated with C–H in-plane bending was located at 1017 cm^−1^ [17]. The intense and broad bands of the cellulose at 1740, 2914, and 3360 cm^−1^ were absent in the spectrum of the KF@PPy composite, implying that the KF surface was entirely covered with PPy [3]. In the FTIR spectrum of the *f*-CNTs, the stretching vibrations of the C=O and C–O bonds of carboxylic acids appeared at 1740 and 1217 cm^−1^, respectively. The absorbance peak at 1365 cm^−1^ was assigned to stretching of conjugated C–O bonds [62]. The band at 3453 cm^−1^ is attributed to the stretching vibrations of the OH groups of carboxyl units [50]. A broad overlapped peak near 2995 cm^−1^ corresponds to asymmetric and symmetric C–H stretching [70]. These results confirm the successful oxidization of the CNTs under the mild conditions. The spectrum of the KF@PPy/*f*-CNT11 composite featured peaks similar to those of KF@PPy, with additional signals at 1217, 1365_,_ 1740, 2995, and 3453 cm^−1^, corresponding to the absorptions of the *f*-CNTs.

**Figure 2:**
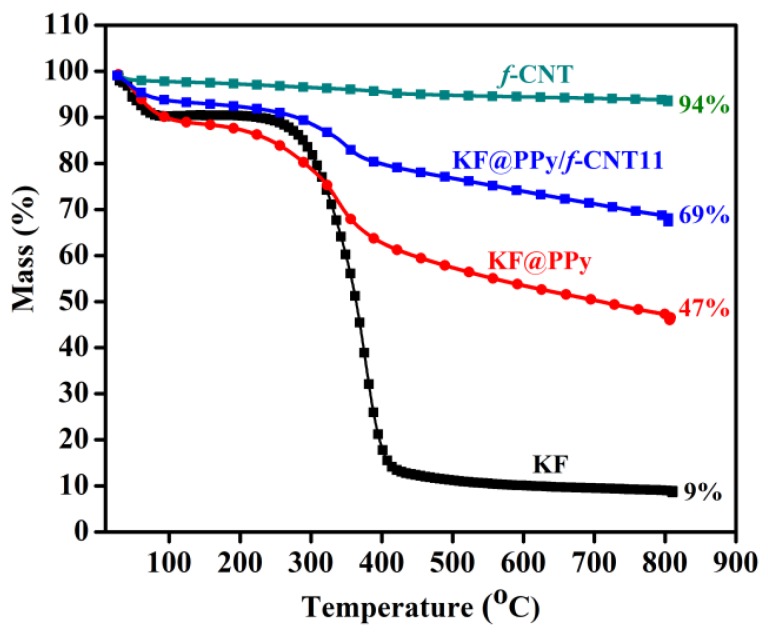
TGA curves of KF, KF@PPy, *f*-CNTs, and KF@PPy/*f*-CNT11.

The thermal stability of the KF, KF@PPy, *f*-CNTs, and KF@PPy/*f*-CNT composite films was examined through TGA under N_2_ over the temperature range 30–800 °C (Figure 2). In the TGA curve of KF, the weight loss during the first stage, from ambient temperature to 100 °C, is assigned to the dehydration of KF, including water molecules that had been absorbed to the KF and those derived from the OH groups [30]. The dramatic weight loss during the second period (280–400 °C) is ascribed to the destruction of the crystalline region of the KF cellulose and amorphous cellulose into d-glucopyranose and free radicals [28]. The KF@PPy composite underwent a much lower weight loss relative to KF, where 47% of the mass was preserved at 800 °C—suggesting good thermal stability for the KF@PPy composite. The TGA curve of the KF@PPy/*f*-CNT11 composite reveals an increased amount of residue when compared with that of the KF@PPy composite, due to the presence of the highly thermally stable *f*-CNTs. The weight residue of the KF@PPy/*f*-CNT11 sample was 69%, indicating its excellent thermal stability under high temperature.

**Figure 3:**
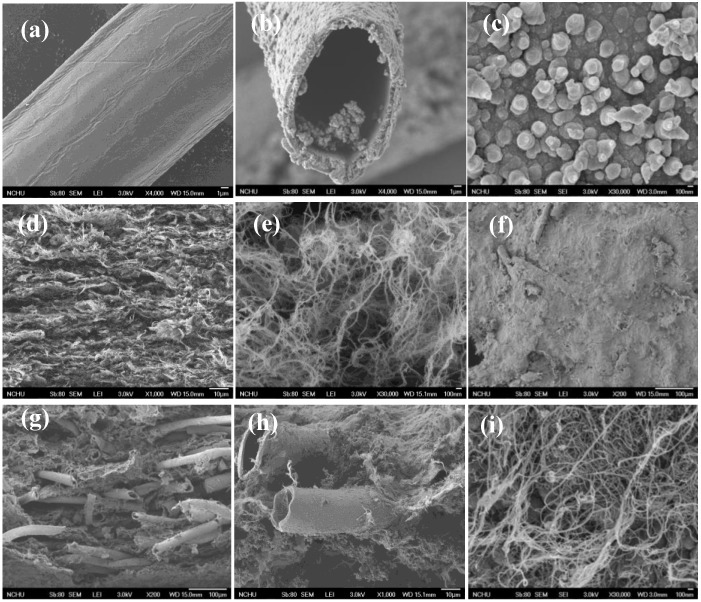
SEM images of (**a**) the pretreated KF; (**b**,**c**) KF@PPy at (**b**) low and (**c**) high magnification; (**d**, **e**) the cross-section of the *f*-CNT freestanding film at (**d**) low and (**e**) high magnification; (**f**) the surface of the KF@PPy/*f*-CNT11 hybrid film at low magnification; and (**g**–**i**) the cross-section of the KF@PPy/*f*-CNT11 hybrid film at (**g**,**h**) low and (**i**) high magnification.

The microstructures and morphologies of the samples were investigated using SEM. As revealed in Figure S1a, the raw KF is characterized by a 3-D hollow microtubular structure, with a smooth surface having an outer diameter of approximately 20–25 µm. This hollow tubular structure with thin walls greatly lowers the density of KF and is highly beneficial for forming ultra-lightweight composites of high surface area. Figure 3a presents the SEM image of the pretreated KF. Although the fiber had retained its hollow structure after pretreatment, the fiber surface became rough, with some wrinkles appearing after the removal of the waxy layer coating the fiber surface. The SEM image of KF@PPy (Figure 3b) confirmed the formation of a conductive backbone of PPy around the inner and outer walls of the KF. This facile and uniform distribution of PPy on the inner and outer walls presumably resulted from the formation of hydrogen bonds between the imino groups of the pyrrole and the large number of OH groups on the KF cellulose. When PPy was synthesized in the absence of KF, PPy-agglomerated particles of large size (Figure S1b) were formed. In the case of KF@PPy, however, the interactions between the NH units of the and the OH groups of the KF cellulose acted as a traction force to support the uniform coating of the PPy, thereby preventing its formation of large agglomerated particles [3,37]. A high-magnification SEM image (Figure 3c) of the KF@PPy surface revealed small nodule-like PPy nanoparticles on the KF surface, instead of the typical cauliflower-like big agglomerates of pure PPy (Figure S1b). These nodular nanostructures would presumably increase the interaction of the PPy with the *f*-CNTs, owing to the increased effective contact surface area. As displayed in Figure 3d,e, the pure *f*-CNT freestanding film possessed a morphology characterized by randomly entangled and cross-linked nanotubular structures that formed a densely packed and compact structure. Figure S2a,b reveal that the mild oxidization conditions helped the *f*-CNTs retain their original length without structural damage. Nanotubes with high-aspect ratios are highly desirable because they can randomly entangle to form freestanding structures, without the aid of any additional polymeric binders. Figure 3f displays the surface of the KF@PPy/*f*-CNT11 hybrid film. Most of the KF@PPy fibers were trapped inside the porous network of *f*-CNTs; only a few were evident on the surface. Figure 3g–i present cross-sectional SEM images of the 3-D porous structured KF@PPy/*f*-CNT11 composite, which consisted of KF@PPy embedded in the interspaces of a porous *f*-CNTs matrix. A high-magnification image (Figure 3i) of the KF@PPy/*f*-CNT11 composite revealed a distribution of the *f*-CNT interconnecting network in a 3-D porous system on the surface of KF@PPy. A strong interfacial interaction was likely to form between the PPy-coated KF and the *f*-CNTs, due to hydrogen bonding. The continuous porous network of *f*-CNTs wrapped around the KF@PPy surface not only acted as a structural reinforcing material but also as a highly conductive skeleton for rapid charge transportation, thereby potentially enhancing the electrochemical performance. Moreover, the highly porous nature of the hybrid film would presumably enhance the interaction of the electrolyte with the electrode materials, leading to better use of the active material for the electrochemical reactions.

**Figure 4:**
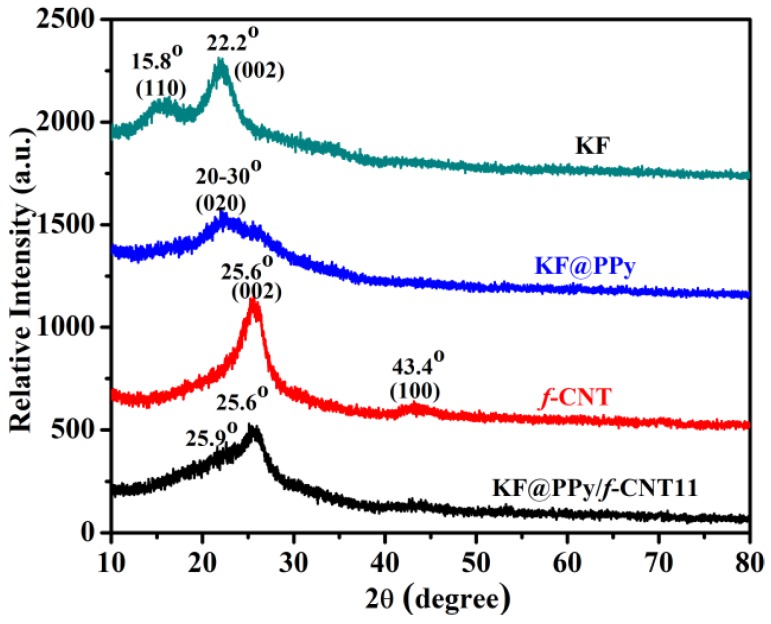
XRD patterns of KF, KF@PPy, *f*-CNTs, and KF@PPy/*f*-CNT11.

Figure 4 presents XRD patterns of the KF, KF@PPy, *f*-CNT, and KF@PPy/*f*-CNT11 samples. The XRD pattern of the KF exhibited two distinct peaks at 2*θ* = 22.2 and 15.8°, corresponding to the (002) and (110) lattice planes, respectively, of cellulose I [68,71]. These peaks represent crystalline and amorphous materials in cellulosic fibers. For the KF@PPy composite, the characteristic diffractions peak of cellulose that appeared at 2*θ* = 15.8° disappeared, and the intensity of the diffraction peak at 2*θ* = 22.2° became weak, whereas a new broad peak for PPy appeared at 2*θ* = 20–30°, consistent with the successful coating of PPy on the KF surface [47]. The XRD pattern of the *f*-CNTs featured one major diffraction peak at 2*θ* = 25.6°, corresponding to the (002) lattice planes of the hexagonal graphite structure, and another, lower-intensity peak at 43.4° corresponding to the diffractions of the graphitic planes (110) and (100) collectively, suggesting that the *f*-CNTs were of high purity [68,72]. The sharpness of the peak at 2*θ* = 25.6° indicates that no significant damage occurred to the graphitic structure of the CNTs during the mild oxidization treatment [62]. In the XRD pattern of the KF@PPy/*f*-CNT11 hybrid film, the diffraction peak at 2*θ* = 25.6° was remarkably broad, because the peak of KF@PPy merged with that of the *f*-CNTs. The pattern of KF@PPy featured a broad peak near 2*θ* = 20–30°, related to the (020) plane of the PPy polymer backbone and indicating the amorphous nature of the PPy [47,68]. This peak also appeared in the XRD spectra of the KF@PPy/*f*-CNT11 hybrid film. Thus, the XRD pattern of the KF@PPy/*f*-CNT11 hybrid featured all of the characteristic peaks of both *f*-CNTs and KF@PPy, confirming the successful integration of the KF@PPy and *f*-CNTs in the hybrid film.

**Figure 5:**
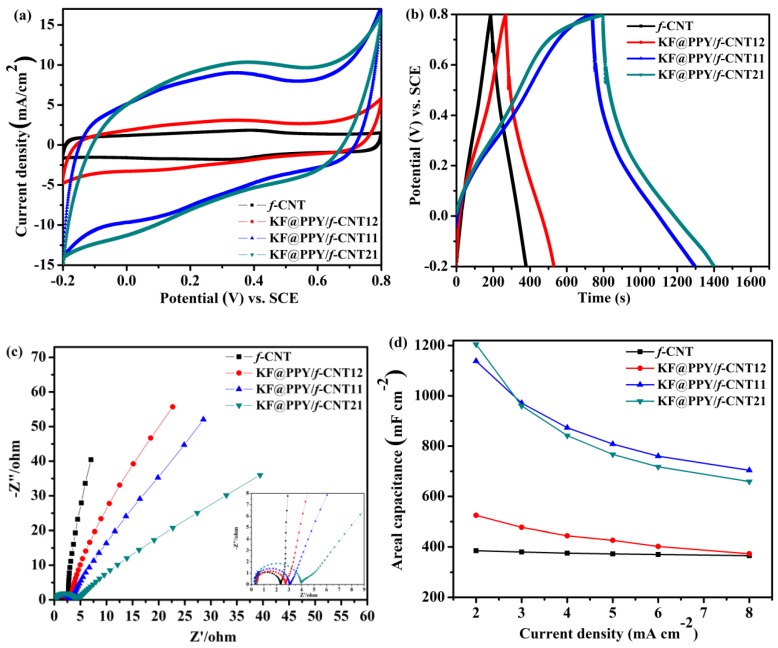
Electrochemical characterization of pure *f*-CNT and KF@PPy/*f*-CNT freestanding hybrid electrodes prepared at various weight ratios (1:2, 1:1, 2:1): (**a**) cyclic voltammetry (CV) curves (scan rate: 5 mV s^−1^); (**b**) galvanostatic charge/discharge (GCD) curves (current density: 2 mA cm^−2^); (**c**) Nyquist plots (frequency range: from 0.01 Hz to 100 kHz), and (**d**) capacitance retention at various current densities.

The KF@PPy/*f*-CNT hybrid films fabricated without the addition of any binder or electroactive additives were used directly as freestanding electrodes to investigate their electrochemical performances in a three-electrode system. For comparison, a pure *f*-CNT freestanding electrode was also tested under the same conditions. Figure 5a presents the CV curves of the *f*-CNT, KF@PPy/*f*-CNT12, KF@PPy/*f*-CNT11, and KF@PPy/*f*-CNT21 freestanding electrodes, measured at a scan rate of 5 mV s^−1^ within a potential window ranging from −0.2 to +0.8 V. The capacitances of the KF@PPy/*f*-CNT freestanding electrodes were much higher than that of the pure *f*-CNT freestanding electrode, as demonstrated by larger enclosed areas in the CV curves. The greater performance of the KF@PPy/*f*-CNT freestanding electrodes can be explained by the synergetic effect of the highly pseudocapacitive PPy and the highly conductive *f*-CNT, resulting in an enhancement in capacitance for the hybrid electrodes. The pure *f*-CNT freestanding electrode exhibited a rectangular CV curve, typical electrical double-layer capacitance (EDLC) behavior of an electrode, with a pair of redox peaks located near 0.4 V (Figure S3). These redox peaks were due to the oxygen-containing groups in the *f*-CNTs that can give rise to a slight pseudocapacitance [73]. The presence of these redox peaks confirmed the effectiveness of the mild oxidization conditions for functionalization of the *f*-CNTs. CV of the KF@PPy/*f*-CNT freestanding electrodes resulted in distorted rectangular curves, due to the contributions to the capacitance arising from the pseudocapacitance of PPy and the EDL capacitance of the *f*-CNTs. Figure 5b presents the GCD curves of the *f*-CNT and KF@PPy/*f*-CNT freestanding electrodes, measured at a current density of 2 mA cm^−2^. The GCD curve of the *f*-CNT freestanding electrode had a relatively symmetrical triangular shape, suggesting ideal EDL capacitive behavior; that of the KF@PPy/*f*-CNT freestanding electrodes exhibited a slight deviation from the symmetrical triangular shape, owing to the contribution to charge storage of the pseudocapacitive PPy. Although the PPy loading of the KF@PPy/*f*-CNT21 hybrid electrode was higher than that of the KF@PPy/*f*-CNT11 hybrid electrode, the increase in areal capacitance, as indicated by the increase in discharge time, was negligibly small, due to the increased charge transfer resistance and diffusion resistance of the KF@PPy/*f*-CNT21 hybrid electrode (as shown in the EIS data, Figure 5c). This behavior is in agreement with the CV results, marked by a small difference between the enclosed areas of the KF@PPy/*f*-CNT11 and KF@PPy/*f*-CNT21 hybrid electrodes. The areal capacitances of the KF@PPy/*f*-CNT11 and KF@PPy/*f*-CNT21 freestanding hybrid electrodes, calculated from the CV curves, at the same current density of 5 mV s^−1^ were 1289 and 1362 mF cm^−2^, respectively. In addition to a high areal capacitance, a high volume-specific capacitance is also a crucial requirement for SC electrodes if they are to find practical applications. The calculated volumetric capacitances of the KF@PPy/*f*-CNT11 and KF@PPy/*f*-CNT21 freestanding hybrid electrodes were 52 and 57 F cm^−3^, respectively. The CV curves recorded at various scan rates and the GCD curves recorded at various current densities of the pure *f*-CNT, KF@PPy/*f*-CNT12, KF@PPy/*f*-CNT11, and KF@PPy/*f*-CNT21 freestanding electrodes are presented in Figures S4,5, 6a,b, and S6, respectively. The pure *f*-CNT freestanding electrode and KF@PPy/*f*-CNT12 freestanding hybrid electrode provided near-rectangular CV curves and symmetrical triangular GCD curves because their capacitances were governed mainly by the EDLCs. In contrast, the KF@PPy/*f*-CNT11 and KF@PPy/*f*-CNT21 freestanding hybrid electrodes provided distorted rectangular CV curves and nonlinear GCD curves, indicating the presence of both EDL and pseudocapacitance behavior. The areal capacitances of the freestanding electrodes at various current densities were calculated from the GCD curves (Figure 5d). The areal capacitances of all of the hybrid electrodes decreased upon increasing the current density, due to inefficient utilization of the inner structures of the active materials for charge storage at high current densities. The KF@PPy/*f*-CNT21 hybrid electrode displayed the highest areal capacitance (ca. 1205 mF cm^−2^) at a lower current density of 2 mA cm^−2^, due to its high PPy content. Nevertheless, a rapid decay in its areal capacitance, with only 54% retention, occurred when the current density was increased to 8 mA cm^−2^, because of the increased charge transfer resistance and electrolyte diffusion resistance. On the other hand, the KF@PPy/*f*-CNT12 hybrid electrode displayed an excellent rate capability, with approximately 71% capacitance retention at 8 mA cm^−2^, but its areal capacitance was very low (525 mF cm^–2^) at 2 mA cm^–2^, due to its low PPy content. Based on these results, the KF@PPy/*f*-CNT11 hybrid electrode—with its high areal capacitance (ca. 1138 mF cm^−2^ at 2 mA cm^−2^) and acceptable capacitance retention of 62% at high current densities—as appears to be a promising freestanding electrode, with superior electrochemical properties, for use in all-solid-state SCs. This capacitance is much higher than that of the pure *f*-CNT freestanding electrode (385 mA cm^−2^) at the same current density. The areal/volumetric capacitance of the KF@PPy/*f*-CNT11 hybrid electrode is much higher than those of many previously reported freestanding electrodes for all-solid-state SCs (see Table S1).

EIS was applied to further understand the electrochemical properties of the *f*-CNT and KF@PPy/*f*-CNT freestanding electrodes. Nyquist plots (Figure 5c) of all the freestanding electrodes featured a semicircle in the high-frequency range and a straight line in the low-frequency range. The semicircle at higher frequency corresponds to the charge transfer resistance (*R*_c_), which arises mainly from the sheet resistance of the electrodes and the electrolytic resistance. The linear part in the low-frequency region represents the diffusion-limited electron transfer process. The intercept of the semicircle with the real axis in the high-frequency region represents the solution resistance (*R*_s_), arising from the electrolyte. The linear part in the plot of the pure *f*-CNT electrode in the low-frequency region is more parallel to the imaginary axis, indicating its high conductivity and pure EDL capacitive behavior. The plot of the KF@PPy/*f*-CNT freestanding electrodes features lines with an inclined angle against the real axis, corresponding to the redox reaction of PPy giving rise to pseudocapacitance. The plots of the *f*-CNT (2.3 Ω), KF@PPy/*f*-CNT12 (2.7 Ω), and KF@PPy/*f*-CNT11 (3.0 Ω) freestanding electrodes each featured a small semicircle in the high-frequency range, suggesting low charge transfer resistance and good conductivity for the KF@PPy/*f*-CNT12 and KF@PPy/*f*-CNT11 hybrid electrodes. In general, the conductivity of *f*-CNT is higher than that of PPy. The less *R*_c_ value and low resistance at low frequency of KF@PPy/*f*-CNT12 freestanding electrode can be ascribed to the overall high conductivity of the composite material resulting from the introduction of 70 wt % *f*-CNTs. However, the EDL capacitance of *f*-CNTs is much less than the pseudocapacitance of PPy. Consequently a high content of *f*-CNT is not favorable for the supercapacitance of freestanding electrode. In addition, the larger values of *R*_s_ and *R*_c_ for the KF@PPy/*f*-CNT21 freestanding electrode (0.41 and 4.0 Ω, respectively) in the high-frequency range may be ascribed to the decrease in conductivity due to the lower content of *f*-CNT, which increased the charge transfer resistance. The EIS data confirmed that the KF@PPy/*f*-CNT11 freestanding electrode had superior electrochemical properties, with low charge transfer resistance and rapid ion diffusion, presumably because of its optimal mass loadings of PPy and *f*-CNT. Accordingly, the KF@PPy/*f*-CNT11 freestanding electrode was used to fabricate an all-solid-state symmetric SC, to investigate its potential in practical applications.

**Figure 6:**
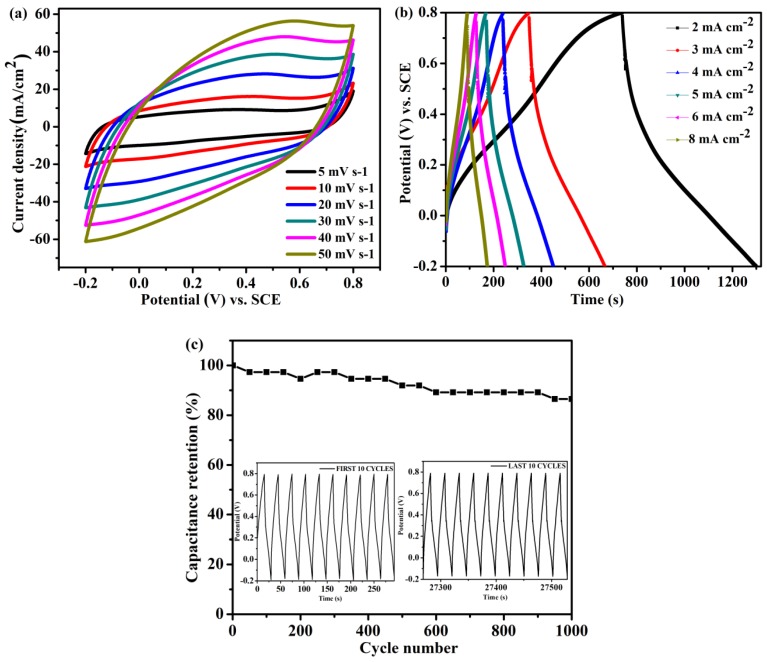
(**a**) CV curves recorded at various scan rates, (**b**) GCD curves recorded at various current densities, and (**c**) cycling life stability after 1000 cycles for the KF@PPy/*f*-CNT11 freestanding hybrid electrode. Inset: First and last 10 cycles.

Figure 6a,b present CV curves recorded at various scan rates and GCD curves recorded at various current densities, respectively, of the KF@PPy/*f*-CNT11 freestanding electrode. The CV curves retained their highly symmetrical shape at all scan rates, indicating the excellent rate capability of the KF@PPy/*f*-CNT11 freestanding electrode. This behavior was confirmed by the GCD curves, which retained similar shapes at all current densities. Moreover, the IR drop of the KF@PPy/*f*-CNT11 freestanding electrode was small, indicative of high conductivity and low charge transfer resistance, even though no additional current collectors were used. Cycling stability is an important parameter for the practical application of any SC in an energy storage device. Most conducting polymers exhibit rapid capacitance decay during repeated charge/discharge processes, due to the large volume change resulting from the rapid redox reactions between the electrode materials and the electrolytes [47]. To evaluate the long-term cycling stability of the KF@PPy/*f*-CNT11 freestanding electrode, a charge/discharge test was performed for 1000 cycles at 25 mA cm^−2^ (Figure 6c). After 1000 cycles, the freestanding electrode retained approximately 86.5% of its initial capacitance, indicating its excellent cycling stability. Thus, the KF cellulose can efficiently accommodate the volume change of the PPy during the charge/discharge cycles.

**Figure 7:**
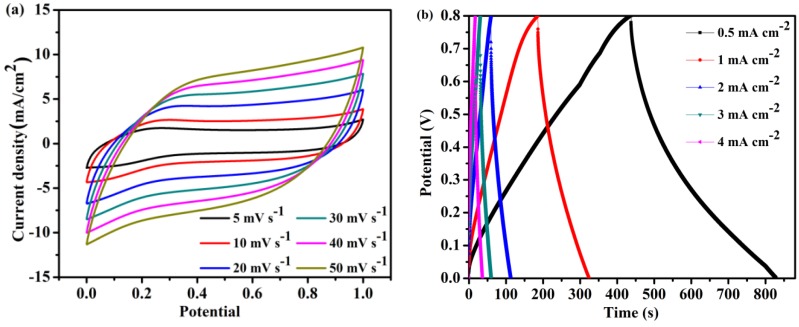

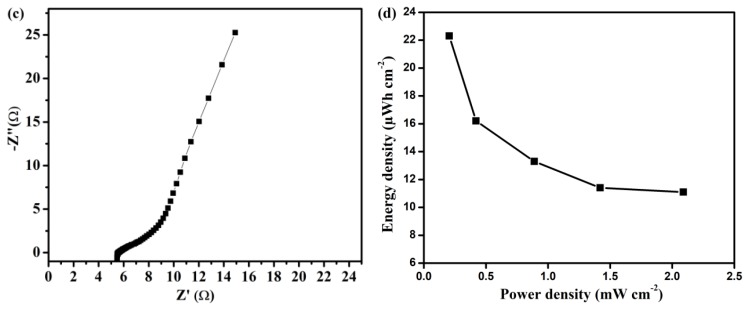
(**a**) CV curves recorded at various scan rates, (**b**) GCD curves recorded at various current densities, (**c**) Nyquist plots recorded over the frequency range from 0.01 Hz to 100 kHz, and (**d**) Ragone plot (power density vs. energy density) of the assembled all-solid-state symmetric SC.

To investigate the potential of using this novel KF@PPy/*f*-CNT11 hybrid freestanding film in practical applications, an all-solid-state symmetric SC was fabricated, featuring this hybrid film as both the positive and negative electrodes and PVA-H_2_SO_4_ gel polymer as both the electrolyte and separator; its electrochemical performance was evaluated in a two-electrode configuration. The gel electrolyte decreased the weight and thickness of the SC considerably, and simplified the device fabrication process—because it did not require any costly special packaging to prevent the leakage of harmful liquid electrolytes. The whole device had a thickness of only approximately 750 µm and a total weight of approximately 102 mg (including the gel electrolyte). The electrochemical properties of the device were determined using CV, GCD, and EIS. Figure 7a presents CV curves of the all-solid-state symmetric SC measured at various scan rates from 5 to 100 mV s^−1^. The CV curves had slightly distorted rectangular shapes because the energy storage process involved a combination of EDL capacitance (*f*-CNT) and pseudocapacitance (PPy). They retained their highly symmetrical shape upon increasing the scan rates, suggesting superior rate performance. The areal capacitances of the symmetrical device, calculated from the CV curves, were 258 and 113 mF cm^−2^ at 5 and 50 mV s^−1^, respectively—that is, the fabricated device displayed good rate capability. Taking into account the total volume of the device, the corresponding volumetric capacitance of the device reached as high as 3.44 F cm^−3^ at 5 mV s^−1^. These excellent electrochemical properties are associated with the unique open porous structure and high electrical conductivity of the hybrid film, facilitating the rapid diffusion and transport of larger ions in the solid electrolyte into the inner surface of the electrode materials. GCD experiments, performed at various current densities, confirmed the superior electrochemical performance of the all-solid-state symmetric SC (Figure 7b). The apparent deviation from linear behavior in the charge–discharge plots is consistent with the CV data, revealing the involvement of faradaic redox reactions in the charge storage process. The discharge curves featured a small IR drop at lower discharge currents (0.049 V at 0.5 mA cm^−2^), indicative of rapid charge transportation at the electrode/electrolyte interface and, hence, a lower equivalent-series resistance (ESR) for the SC device. A low internal resistance is important for energy-storing devices because less energy will be wasted to produce unwanted heat during the processes of charging and discharging. A slight increase in the IR drop was observed upon increasing the current density, due to the inaccessibility of the electrolyte ions to the internal pores of the electrode at higher currents. The areal capacitance of the all-solid-state symmetric SC, evaluated from the GCD experiment, was approximately 219.4 mF cm^−2^ at a discharge current density of 0.5 mA cm^−2^.

EIS was used to further evaluate the electrochemical performance of the electrode materials of the SC device in the frequency range from 0.01 to 100 kHz, with an AC perturbation of 5 mV; the corresponding Nyquist plot (–Z´´ vs. Z´ plot) is presented in Figure 7c. The intercept of the Nyquist plot with the real axis at high frequency indicates the internal resistance, also known as the solution resistance (*R*_s_), of the SC; it is related to the resistance of the electrolyte, the contact resistance between the electrode and current collector, and the intrinsic resistance of the active material. The calculated value of *R*_s_ of the SC was 5.5 Ω; it is most likely associated with the intrinsic resistance of the gel electrolyte. The charge transfer resistance (*R*_c_), indicated by the diameter of the semicircle in the high-frequency region, was approximately 3 Ω. In the low-frequency range, the Nyquist plot of the SC featured an almost vertical line, indicating near-ideal capacitive behavior because of the faster ion transport at the electrode–electrolyte interface. This excellent ion diffusion in the KF@PPy/*f*-CNT11–based SC was due to the highly porous microstructure formed by the KF@PPy and *f*-CNT interconnected network. The pores in the unique porous structure of the hybrid electrode acted as electrolyte ion reservoirs; they shortened the ion diffusion distance by providing good diffusion channels for electrolyte ions. Thus, the accessibility of electrolyte ions was improved, as well as allowing more efficient utilization of PPy and *f*-CNT. Moreover, the Ragone plot of the assembled all-solid-state symmetric device exhibited (Figure 7d) a maximum areal energy density (volumetric energy density) of 22.3 µW h cm^−2^ (297.3 µW h cm^−3^) at an areal power density (volumetric power density) of 0.21 mW cm^−2^ (2.8 mW cm^−3^), and maintained an energy density of 11.1 µW h cm^−2^ (148 µW h cm^−3^) at a maximum power density of 2.1 mW cm^−2^ (28 mW cm^−3^). These values indicate the superior rate capability of the assembled device and its potential for storing and delivering energy at rapid rates. The areal/volume capacitance, maximum areal/volumetric energy density, and power density of the KF@PPy/*f*-CNT11 SC surpass those achieved for many previously reported all-solid-state symmetric SCs, and even some asymmetric SCs (see Table S2).

**Figure 8:**
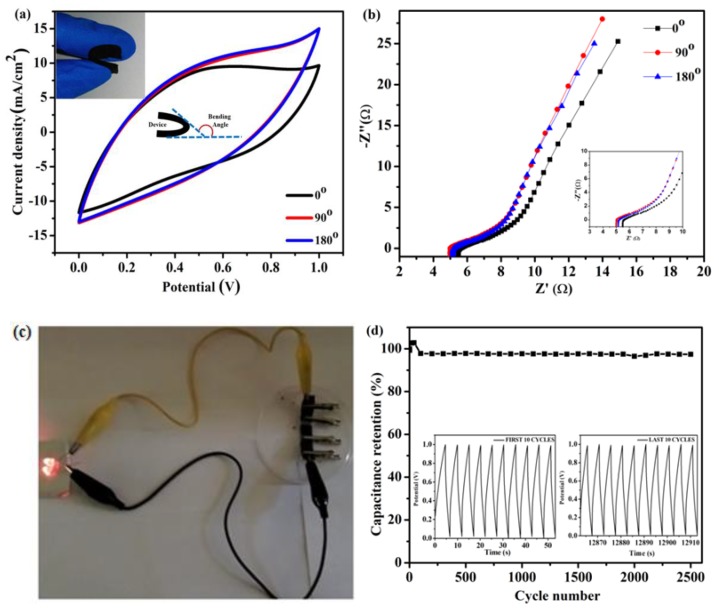
(**a**) CV curves of the KF@PPy/*f*-CNT11 flexible SC subjected to bending at various angles; scan rate: 40 mV s^−1^ (inset: digital photograph of the KF@PPy/*f*-CNT11 flexible SC in a bending state). (**b**) EIS spectra of the KF@PPy/*f*-CNT11 flexible SC at various bending angles. (**c**) Digital photograph of two red LEDs powered by three KF@PPy/*f*-CNT11 flexible SCs connected in series. (**d**) Cycling stability (2500 cycles, 25 mA cm^−2^) of the assembled KF@PPy/*f*-CNT11 flexible all-solid-state symmetric SC.

The flexibility and mechanical stability of the assembled all-solid-state symmetric SC were assessed by bending it from 0° to 180°. As displayed in the inset to Figure 8a, the assembled SC was highly flexible and was readily bent to large angles without causing any mechanical damage. The changes in the electrochemical properties of the SC during the bending process were examined using CV and EIS (Figure 8,b, respectively). Interestingly, in contrast to most previous reports of a slight decrease in the electrical electrochemical properties of the SCs after bending, the electrochemical performance of our SC improved after bending, as indicated by the increase in the CV enclosed area. As a result, the areal capacitance of the device increased from 128.4 mF cm^−2^ at 0° to 137.4 mF cm^−2^ at 90° and to 139.4 mF cm^−2^ at 180°. This increase in the areal capacitance could be explained by a decrease in the contact resistance between the electrode materials and the gel electrolyte, as indicated by the low contact resistance and diffusion resistance in the EIS spectra during the bending process. In addition, the compression of the electrodes of the SC that occurred during the bending process might have acted as an external pressure applied to the device, resulting in increased intimate contact between the PPy and *f*-CNT and, eventually, shortening the distance for charge carrier transportation. In addition, the impedance curves of the SC measured under bending conditions featured a more vertical line parallel to the imaginary axis in the low-frequency region, suggesting improved capacitance behavior. The excellent flexibility of our KF@PPy/*f*-CNT hybrid film–based symmetric SC suggests that it had great promise to be applied in wearable/portable flexible electronics. To demonstrate its practical applications in flexible and portable energy storage systems, three pieces of the as-fabricated flexible all-solid-state SCs were connected in series to power two red LEDs (Figure 8c). Long term cycling stability is a crucial parameter for the practical applications of an all-solid-state SC in portable electronics. To check the electrochemical stability of our device, a GCD test was performed at 25 mA cm^−2^ for 2500 cycles (Figure 8d). The use of the PVA/H_2_SO_4_ gel electrolyte greatly enhanced the cycle life of the all-solid-state KF@PPy/*f*-CNT11 hybrid SC; the capacitance retention was 97.4% after 2500 cycles. The first and last 10 cycles (inset to Figure 8d) provided nearly identical charge discharge curves, confirming the superior cycling stability of the all-solid-state device. The excellent cycling behavior of this solid-state device is ascribed to the highly conductive unique porous structure of the KF@PPy/*f*-CNT11 hybrid electrode, not only ensuring rapid ion transport but also helping to buffer the volumetric change caused by the ion doping and de-doping of the PPy during the charge/discharge process. These results indicate the promising potential of highly conductive and freestanding KF@PPy/*f*-CNT electrodes for use in the fabrication of environmentally friendly, lightweight, inexpensive, highly flexible, high-performance SCs, with applications in portable and flexible electronic gadgets.

## 4. Conclusions

This paper demonstrates a simple and straightforward approach for the fabrication of porous freestanding electrodes, displaying excellent electrochemical properties, and their use in lightweight, inexpensive, and eco-friendly all-solid-state SCs. The as-prepared KF@PPy/*f*-CNT hybrid film could be used as an SC electrode, without the need for any additional current collector, binders, or conducting agents, thereby considerably lowering the total weight and cost of the device. The freestanding composite electrodes exhibited excellent electrochemical performances, due to their unique design and structure. The *f*-CNTs provided a highly conductive network that facilitated electron transfer and improved the mechanical integrity of the composite films. PPy deposited on the hollow KF helped to provide an open porous structure to the KF@PPy/*f*-CNT hybrid films, ensuring easy pathways for the electrolyte ions and high rates of ion transport. The KF also helped to accommodate the PPy volume change during the redox reactions, resulting in excellent cycle stability. A robust all-solid-state symmetric SC, fabricated using the freestanding film, displayed superior capacitive performance. Based on the high areal capacitance and excellent cycle stability, this all-solid-state symmetric SC holds great potential for use in practical applications. The use of KF as an inexpensive and eco-friendly source of cellulose, along with this simple and straightforward synthesis strategy, without requiring any hazardous chemicals or processing conditions, results in a green and bulk-scalable process for the overall fabrication of SCs.

## Supplementary Materials

The following are available online at http://www.mdpi.com/2073-4360/10/11/1247/s1, Figure S1: SEM images of the (**a**) raw Kapok fiber and (**b**) PPy powder, Figure S2: SEM images of the (**a**) raw and (**b**) functionalized CNTs (*f*-CNTs), Figure S3: CV curve of the pure *f*-CNT freestanding electrode, measured at 5 mV s^−1^, Figure S4: (**a**) CV curves recorded at various scan rates and (**b**) GCD curves recorded at various current densities of the pure *f*-CNT freestanding hybrid electrode, Figure S5: (**a**) CV curves recorded at various scan rates and (**b**) GCD curves recorded at various current densities for the KF@PPy/*f*-CNT12 freestanding hybrid electrode, Figure S6: (**a**) CV curves recorded at various scan rates and (**b**) GCD curves recorded at various current densities for the KF@PPy/*f*-CNT21 freestanding hybrid electrode, Table S1: Capacitive performances of freestanding electrodes reported in the literature and in this present study, Table S2: Capacitive performances of all-solid-state supercapacitors reported in the literature and in this study.

## References

[1] Lai H., Wu Q., Zhao J., Shang L., Li H., Che R., Lyu Z., Xiong J., Yang L., Wang X. (2016). Mesostructured Nio/Ni Composites for High-Performance Electrochemical Energy Storage. Energy Environ. Sci..

[2] Li T., Yu H., Zhi L., Zhang W., Dang L., Liu Z., Lei Z. (2017). Facile Electrochemical Fabrication of Porous Fe2o3 Nanosheets for Flexible Asymmetric Supercapacitors. J. Phys. Chem. C.

[3] Wan C., Jiao Y., Li J. (2017). Flexible, Highly Conductive, and Free-Standing Reduced Graphene Oxide/Polypyrrole/Cellulose Hybrid Papers for Supercapacitor Electrodes. J. Mater. Chem. A.

[4] Tao J., Liu N., Li L., Su J., Gao Y. (2014). Hierarchical Nanostructures of Polypyrrole@Mno2 Composite Electrodes for High Performance Solid-State Asymmetric Supercapacitors. Nanoscale.

[5] Koga H., Tonomura H., Nogi M., Suganuma K., Nishina Y. (2016). Fast, Scalable, and Eco-Friendly Fabrication of an Energy Storage Paper Electrode. Green Chem..

[6] Anothumakkool B., Soni R., Bhange S.N., Kurungot S. (2015). Novel Scalable Synthesis of Highly Conducting and Robust Pedot Paper for a High Performance Flexible Solid Supercapacitor. Energy Environ. Sci..

[7] Liu R., Ma L., Huang S., Mei J., Xu J., Yuan G. (2017). A Flexible Polyaniline/Graphene/Bacterial Cellulose Supercapacitor Electrode. New J. Chem..

[8] Yang C., Zhang L., Hu N., Yang Z., Wei H., Zhang Y. (2016). Reduced Graphene Oxide/Polypyrrole Nanotube Papers for Flexible All-Solid-State Supercapacitors with Excellent Rate Capability and High Energy Density. J. Power Sources.

[9] Feng J.X., Ye S.H., Lu X.F., Tong Y.X., Li G.R. (2015). Asymmetric Paper Supercapacitor Based on Amorphous Porous Mn3o4 Negative Electrode and Ni(Oh)2 Positive Electrode: A Novel and High-Performance Flexible Electrochemical Energy Storage Device. ACS Appl. Mater. Interfaces.

[10] Chen Y., Cai K., Liu C., Song H., Yang X. (2017). High-Performance and Breathable Polypyrrole Coated Air-Laid Paper for Flexible All-Solid-State Supercapacitors. Adv. Energy Mater..

[11] Cao Z., Wei B. (2013). A Perspective: Carbon Nanotube Macro-Films for Energy Storage. Energy Environ. Sci..

[12] Zhao J., Li C., Zhang Q., Zhang J., Wang X., Lin Z., Wang J., Lv W., Lu C., Wong C. (2017). An All-Solid-State, Lightweight, and Flexible Asymmetric Supercapacitor Based on Cabbage-Like Znco2o4 and Porous Vn Nanowires Electrode Materials. J. Mater. Chem. A.

[13] Yao B., Yuan L., Xiao X., Zhang J., Qi Y., Zhou J., Zhou J., Hu B., Chen W. (2013). Paper-Based Solid-State Supercapacitors with Pencil-Drawing Graphite/Polyaniline Networks Hybrid Electrodes. Nano Energy.

[14] Chen Z., Liao W., Ni X. (2017). Spherical Polypyrrole Nanoparticles Growing on the Reduced Graphene Oxide-Coated Carbon Cloth for High Performance and Flexible All-Solid-State Supercapacitors. Chem. Eng. J..

[15] Lee D., Cho Y.G., Song H.K., Chun S.J., Park S.B., Choi D.H., Lee S.Y., Yoo J., Lee S.Y. (2017). Coffee-Driven Green Activation of Cellulose and Its Use for All-Paper Flexible Supercapacitors. ACS Appl. Mater. Interfaces.

[16] Liew S.Y., Walsh D.A., Thielemans W. (2013). High Total-Electrode and Mass-Specific Capacitance Cellulose Nanocrystal-Polypyrrole Nanocomposites for Supercapacitors. RSC Adv..

[17] Wang F., Kim H.J., Park S., Kee C.D., Kim S.J., Oh I.K. (2016). Bendable and Flexible Supercapacitor Based on Polypyrrole-Coated Bacterial Cellulose Core-Shell Composite Network. Compos. Sci. Technol..

[18] Peng S., Fan L., Wei C., Liu X., Zhang H., Xu W., Xu J. (2017). Flexible Polypyrrole/Copper Sulfide/Bacterial Cellulose Nanofibrous Composite Membranes as Supercapacitor Electrodes. Carbohydr. Polym..

[19] Shi Z., Phillips G.O., Yang G. (2013). Nanocellulose Electroconductive Composites. Nanoscale.

[20] Ge D., Yang L., Fan L., Zhang C., Xiao X., Gogotsi Y., Yang S. (2015). Foldable Supercapacitors from Triple Networks of Macroporous Cellulose Fibers, Single-Walled Carbon Nanotubes and Polyaniline Nanoribbons. Nano Energy.

[21] Hu L., Pasta M., la Mantia F., Cui L., Jeong S., Deshazer H.D., Choi J.W., Han S.M., Cui Y. (2010). Stretchable, Porous, and Conductive Energy Textiles. Nano Lett..

[22] Su H., Zhu P., Zhang L., Zeng W., Zhou F., li G., Li T., Wang Q., Sun R., Wong C. (2016). Low Cost, High Performance Flexible Asymmetric Supercapacitor Based on Modified Filter Paper and an Ultra-Fast Packaging Technique. RSC Adv..

[23] Ma L., Liu R., Niu H., Zhao M., Huang Y. (2016). Flexible and Freestanding Electrode Based on Polypyrrole/Graphene/Bacterial Cellulose Paper for Supercapacitor. Compos. Sci. Technol..

[24] Weng Z., Su Y., Wang D., Li F., Du J., Cheng H.M. (2011). Graphene–Cellulose Paper Flexible Supercapacitors. Adv. Energy Mater..

[25] Yuan L., Yao B., Hu B., Huo K., Chen W., Zhou J. (2013). Polypyrrole-Coated Paper for Flexible Solid-State Energy Storage. Energy Environ. Sci..

[26] Wu X., Chabot V.L., Kim B.K., Yu A., Berry R.M., Tam K.C. (2014). Cost-Effective and Scalable Chemical Synthesis of Conductive Cellulose Nanocrystals for High-Performance Supercapacitors. Electrochim. Acta.

[27] Zhang L., Yu X., Zhu P., Zhou F., Li G., Sun R., Wong C.P. (2017). Laboratory Filter Paper as a Substrate Material for Flexible Supercapacitors. Sustain. Energy Fuels.

[28] Peng S., Xu Q., Fan L., Wei C., Bao H., Xu W., Xu J. (2016). Flexible Polypyrrole/Cobalt Sulfide/Bacterial Cellulose Composite Membranes for Supercapacitor Application. Synth. Metals.

[29] Jost K., Perez C.R., McDonough J.K., Presser V., Heon M., Dion G., Gogotsi Y. (2011). Carbon Coated Textiles for Flexible Energy Storage. Energy Environ. Sci..

[30] Liu C., Cai Z., Zhao Y., Zhao H., Ge F. (2016). Potentiostatically Synthesized Flexible Polypyrrole/Multi-Wall Carbon Nanotube/Cotton Fabric Electrodes for Supercapacitors. Cellulose.

[31] Liang G., Zhu L., Xu J., Fang D., Bai Z., Xu W. (2013). Investigations of Poly(Pyrrole)-Coated Cotton Fabrics Prepared in Blends of Anionic and Cationic Surfactants as Flexible Electrode. Electrochim. Acta.

[32] Wang Z., Tammela P., Zhang P., Huo J., Ericson F., Stromme M., Nyholm L. (2014). Freestanding Nanocellulose-Composite Fibre Reinforced 3d Polypyrrole Electrodes for Energy Storage Applications. Nanoscale.

[33] Yao B., Zhang J., Kou T., Song Y., Liu T., Li Y. (2017). Paper-Based Electrodes for Flexible Energy Storage Devices. Adv. Sci..

[34] Lv S., Fu F., Wang S., Huang J., Hu L. (2015). Novel Wood-Based All-Solid-State Flexible Supercapacitors Fabricated with a Natural Porous Wood Slice and Polypyrrole. RSC Adv..

[35] Zhu H., Luo W., Ciesielski P.N., Fang Z., Zhu J.Y., Henriksson G., Himmel M.E., Hu L. (2016). Wood-Derived Materials for Green Electronics, Biological Devices, and Energy Applications. Chem. Rev..

[36] Perez-Madrigal M.M., Edo M.G., Aleman C. (2016). Powering the Future: Application of Cellulose-Based Materials for Supercapacitors. Green Chem..

[37] Xu J., Zhu L., Bai Z., Liang G., Liu L., Fang D., Xu W. (2013). Conductive Polypyrrole–Bacterial Cellulose Nanocomposite Membranes as Flexible Supercapacitor Electrode. Org. Electron..

[38] Tsouko E., Kourmentza C., Ladakis D., Kopsahelis N., Mandala I., Papanikolaou S., Paloukis F., Alves V., Koutinas A. (2015). Bacterial Cellulose Production from Industrial Waste and by-Product Streams. Int. J. Mol. Sci..

[39] Huang S., Chen P., Lin W., Lyu S., Chen G., Yin X., Chen W. (2016). Electrodeposition of Polypyrrole on Carbon Nanotube-Coated Cotton Fabrics for All-Solid Flexible Supercapacitor Electrodes. RSC Adv..

[40] Xu W., Mu B., Wang A. (2017). Morphology Control of Polyaniline by Dopant Grown on Hollow Carbon Fibers as High-Performance Supercapacitor Electrodes. Cellulose.

[41] Zheng Y., Wang A., Hakeem K.R., Jawaid M., Rashid U. (2014). Kapok Fiber: Structure and Properties. Biomass and Bioenergy: Processing and Properties.

[42] Xu W., Mu B., Zhang W., Wang A. (2016). Facile Fabrication of Well-Defined Polyaniline Microtubes Derived from Natural Kapok Fibers for Supercapacitors with Long-Term Cycling Stability. RSC Adv..

[43] Xu W., Mu B., Wang A. (2017). Three-Dimensional Hollow Microtubular Carbonized Kapok Fiber/Cobalt-Nickel Binary Oxide Composites for High-Performance Electrode Materials of Supercapacitors. Electrochim. Acta.

[44] Xu W., Mu B., Zhang W., Wang A. (2015). Facile Hydrothermal Synthesis of Tubular Kapok Fiber/Mno2 Composites and Application in Supercapacitors. RSC Adv..

[45] Foulk J.A., Chao W.Y., Akin D.E., Dodd R.B., Layton P.A. (2006). Analysis of Flax and Cotton Fiber Fabric Blends and Recycled Polyethylene Composites. J. Polym. Environ..

[46] Lee K.Y., Blaker J.J., Bismarck A. (2009). Surface Functionalisation of Bacterial Cellulose as the Route to Produce Green Polylactide Nanocomposites with Improved Properties. Compos. Sci. Technol..

[47] Muhamad S.U., Idris N.H., Yusoff H.M., Din M.F.M., Majid S.R. (2017). In-Situ Encapsulation of Nickel Nanoparticles in Polypyrrole Nanofibres with Enhanced Performance for Supercapacitor. Electrochim. Acta.

[48] Chen Y., Du L., Yang P., Sun P., Yu X., Mai W. (2015). Significantly Enhanced Robustness and Electrochemical Performance of Flexible Carbon Nanotube-Based Supercapacitors by Electrodepositing Polypyrrole. J. Power Sources.

[49] Zhou H., Zhai H.J. (2016). A Highly Flexible Solid-State Supercapacitor Based on the Carbon Nanotube Doped Graphene Oxide/Polypyrrole Composites with Superior Electrochemical Performances. Org. Electron..

[50] Liu P., Wang X., Li H. (2013). Preparation of Carboxylated Carbon Nanotubes/Polypyrrole Composite Hollow Microspheres Via Chemical Oxidative Interfacial Polymerization and Their Electrochemical Performance. Synth. Metals.

[51] Cai X., Hansen R.V., Zhang L., Li B., Poh C.K., Lim S.H., Chen L., Yang J., Lai L., Lin J. (2015). Binary Metal Sulfides and Polypyrrole on Vertically Aligned Carbon Nanotube Arrays/Carbon Fiber Paper as High-Performance Electrodes. J. Mater. Chem. A.

[52] Wei H., Wang Y., Guo J., Yan X., O’Connor R., Zhang X., Shen N.Z., Weeks B.L., Huang X., Wei S. (2015). Electropolymerized Polypyrrole Nanocoatings on Carbon Paper for Electrochemical Energy Storage. ChemElectroChem.

[53] Yang C., Shen J., Wang C., Fei H., Bao H., Wang G. (2014). All-Solid-State Asymmetric Supercapacitor Based on Reduced Graphene Oxide/Carbon Nanotube and Carbon Fiber Paper/Polypyrrole Electrodes. J. Mater. Chem. A.

[54] Feng D.Y., Song Y., Huang Z.H., Xu X.X., Liu X.X. (2016). Rate Capability Improvement of Polypyrrole Via Integration with Functionalized Commercial Carbon Cloth for Pseudocapacitor. J. Power Sources.

[55] Liu T., Finn L., Yu M., Wang H., Zhai T., Lu X., Tong Y., Li Y. (2014). Polyaniline and Polypyrrole Pseudocapacitor Electrodes with Excellent Cycling Stability. Nano Lett..

[56] Yesi Y., Shown I., Ganguly A., Ngo T.T., Chen L., Chen K. (2016). Directly-Grown Hierarchical Carbon Nanotube@Polypyrrole Core–Shell Hybrid for High-Performance Flexible Supercapacitors. ChemSusChem.

[57] Santino L.M., Acharya S., D’Arcy J.M. (2017). Low-Temperature Vapour Phase Polymerized Polypyrrole Nanobrushes for Supercapacitors. J. Mater. Chem. A.

[58] Cong H.P., Ren X.C., Wang P., Yu S.H. (2013). Flexible Graphene-Polyaniline Composite Paper for High-Performance Supercapacitor. Energy Environ. Sci..

[59] Liu Y., Wang J., Zheng Y., Wang A. (2012). Adsorption of Methylene Blue by Kapok Fiber Treated by Sodium Chlorite Optimized with Response Surface Methodology. Chem. Eng. J..

[60] Avilés F., Cauich-Rodríguez J.V., Moo-Tah L., May-Pat A., Vargas-Coronado R. (2009). Evaluation of Mild Acid Oxidation Treatments for Mwcnt Functionalization. Carbon.

[61] Datsyuk V., Kalyva M., Papagelis K., Parthenios J., Tasis D., Siokou A., Kallitsis I., Galiotis C. (2008). Chemical Oxidation of Multiwalled Carbon Nanotubes. Carbon.

[62] Saleh T.A. (2011). The Influence of Treatment Temperature on the Acidity of Mwcnt Oxidized by Hno3 or a Mixture of Hno3/H2so4. Appl. Surf. Sci..

[63] Reddy K.R., Sin B.C., Ryu K.S., Kim J.C., Chung H., Lee Y. (2009). Conducting Polymer Functionalized Multi-Walled Carbon Nanotubes with Noble Metal Nanoparticles: Synthesis, Morphological Characteristics and Electrical Properties. Synth. Metals.

[64] Fu H., Du Z.J., Zou W., Li H.Q., Zhang C. (2013). Carbon Nanotube Reinforced Polypyrrole Nanowire Network as a High-Performance Supercapacitor Electrode. J. Mater. Chem. A.

[65] Xu L., Jia M., Li Y., Zhang S., Jin X. (2017). Design and Synthesis of Graphene/Activated Carbon/Polypyrrole Flexible Supercapacitor Electrodes. RSC Adv..

[66] Shu K., Wang C., Zhao C., Ge Y., Wallace G.G. (2016). A Free-Standing Graphene-Polypyrrole Hybrid Paper Via Electropolymerization with an Enhanced Areal Capacitance. Electrochim. Acta.

[67] Xu J., Wang D., Yuan Y., Wei W., Duan L., Wang L., Bao H., Xu W. (2015). Polypyrrole/Reduced Graphene Oxide Coated Fabric Electrodes for Supercapacitor Application. Org. Electron..

[68] Li N., Li X., Yang C., Wang F., Li J., Wang H., Chen C., Liu S., Pan Y., Li D. (2016). Fabrication of a Flexible Free-Standing Film Electrode Composed of Polypyrrole Coated Cellulose Nanofibers/Multi-Walled Carbon Nanotubes Composite for Supercapacitors. RSC Adv..

[69] Yang C., Li D. (2015). Flexible and Foldable Supercapacitor Electrodes from the Porous 3d Network of Cellulose Nanofibers, Carbon Nanotubes and Polyaniline. Mater. Lett..

[70] Zhang X., Tao L., He P., Zhang X., He M., Dong F., He S., Li C., Liu H., Wang S. (2018). A Novel Cobalt Hexacyanoferrate/Multi-Walled Carbon Nanotubes Nanocomposite: Spontaneous Assembly Synthesis and Application as Electrode Materials with Significantly Improved Capacitance for Supercapacitors. Electrochim. Acta.

[71] Lyu S., Chang H., Fu F., Hu L., Huang J., Wang S. (2016). Cellulose-Coupled Graphene/Polypyrrole Composite Electrodes Containing Conducting Networks Built by Carbon Fibers as Wearable Supercapacitors with Excellent Foldability and Tailorability. J. Power Sources.

[72] Tang L., Yang Z., Duan F., Chen M. (2017). Hierarchical Architecture of Ultrashort Carbon Nanotubes/Polyaniline Nanocables Coated on Graphene Sheets for Advanced Supercapacitors. J. Mater. Sci. Mater. Electron..

[73] Xiao X., Li T., Peng Z., Jin H., Zhong Q., Hu Q., Yao B., Luo Q., Zhang C., Gong L. (2014). Freestanding Functionalized Carbon Nanotube-Based Electrode for Solid-State Asymmetric Supercapacitors. Nano Energy.

