# Supplementary material for: Facile, Scalable, Eco-Friendly Fabrication of High-Performance Flexible All-Solid-State Supercapacitors
Source: Polymers (Basel). 2018 Nov 11;10(11):1247. doi: 10.3390/polym10111247 (PMC6401692; doi:10.3390/polym10111247)
Supplement: Supplementary file 1 [file polymers-10-01247-s001.pdf]

Supplementary Information

Facile, scalable, eco-friendly fabrication of high-performance flexible all-solid-state supercapacitors

Jincy Parayangattil Jyothibasu [1,2] and Rong-Ho Lee [2,*]

[1] Department of Environmental Engineering, National Chung Hsing University, Taichung 402, Taiwan ; jincycusat@gmail.com

[2] Department of Chemical Engineering, National Chung Hsing University, Taichung 402, Taiwan; rhl@nchu.edu.tw

* Correspondence: Rong-Ho Lee—e-mail: rhl@dragon.nchu.edu.tw; Tel.: +886-4-22854308

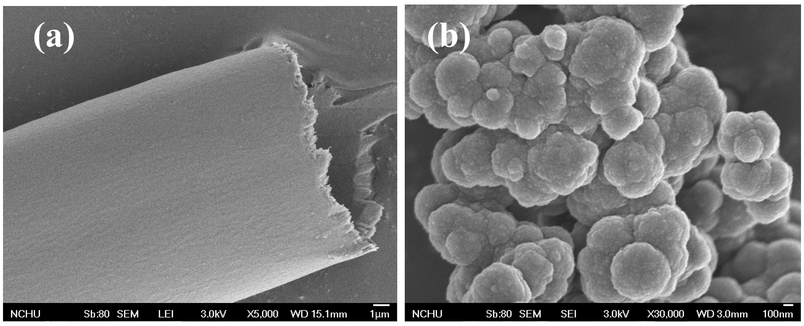

Fig. S1 SEM images of the (a) raw Kapok fiber and (b) PPy powder.

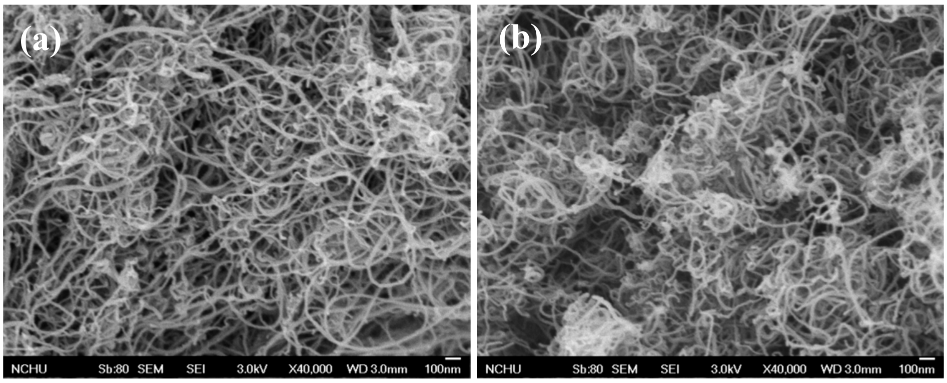

Fig. S2 SEM images of the (a) raw and (b) functionalized CNTs (*f*-CNTs).

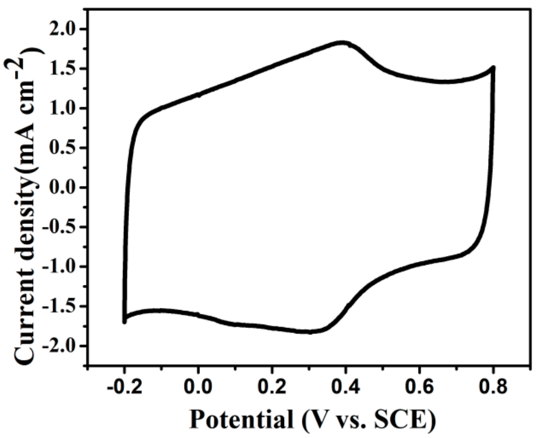

Fig. S3 CV curve of the pure *f*-CNT freestanding electrode, measured at 5 mV s^{-1}.

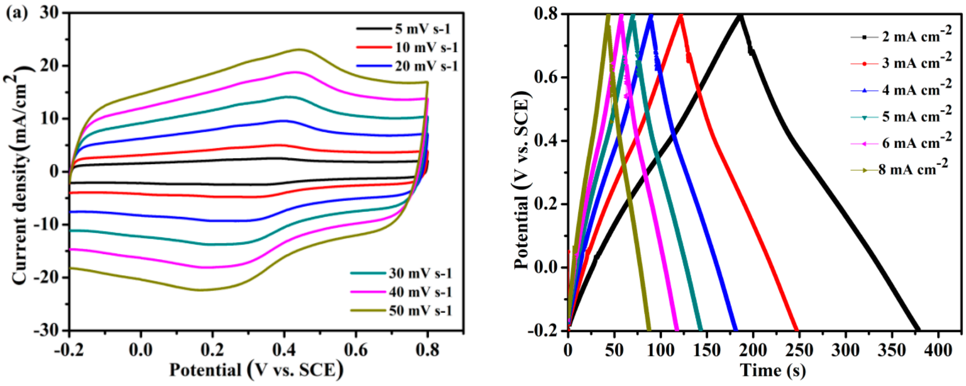

Fig. S4 (a) CV curves recorded at various scan rates and (b) GCD curves recorded at various current densities of the pure *f*-CNT freestanding hybrid electrode.

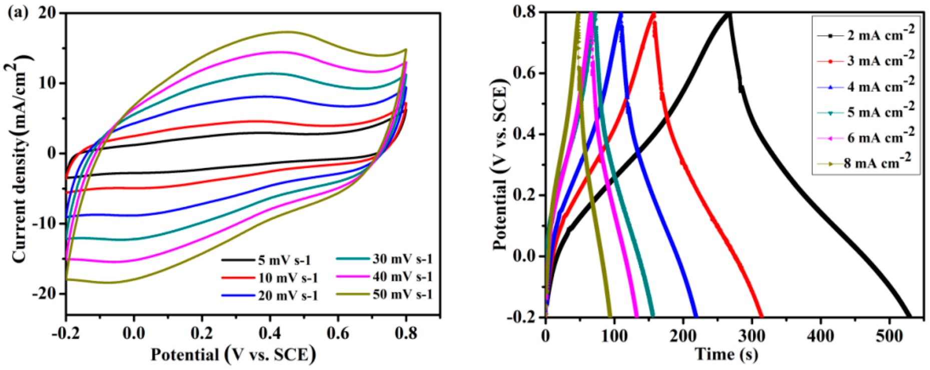

Fig. S5 (a) CV curves recorded at various scan rates and (b) GCD curves recorded at various current densities for the KF@PPy/*f*-CNT12 freestanding hybrid electrode.

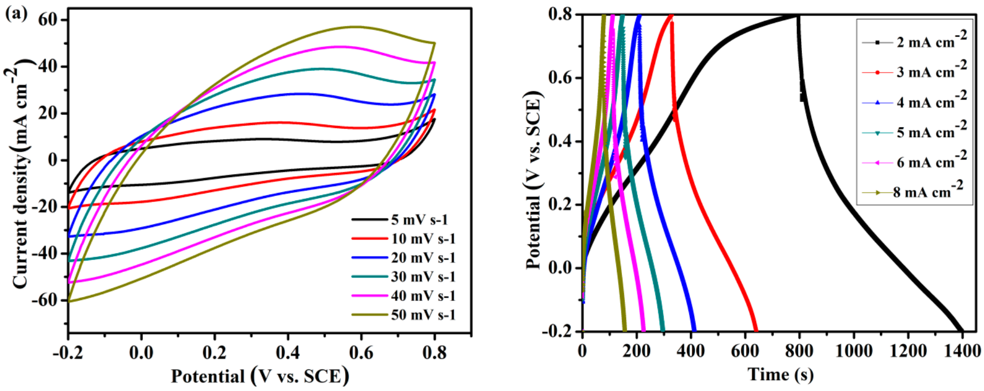

Fig. S6 (a) CV curves recorded at various scan rates and (b) GCD curves recorded at various current densities for the KF@PPy/*f*-CNT21 freestanding hybrid electrode.

TABLE S1 Capacitive performances of freestanding electrodes reported in the literature and in this present study

Electrode materials	Specific capacitance of electrode	Electrolyte	Capacitance retention	Ref.
**KF@PPy/*f*-CNT11**	**1289 mF cm^{-2} (5 mV s^{-1})**<br>**1138 mF cm^{-2} (2 mA cm^{-2})**<br>**52 F cm^{-3} (5 mV s^{-1})**	**1 M H_2SO_4**	**86.5% (1000 cycles)**	**This work**
rGO/PPy NT paper	807 mF cm^{-2} (1 mA cm^{-2})	1 M H_2SO_4	78.0% (2000 cycles)	**1**
GO-PPy paper	440 mF cm^{-2} (0.5 A g^{-1})	1 M H_2SO_4	81%	**2**
rGO/PPy paper	468 mF cm^{-2} (1 mA cm^{-2})	1 M H_2SO_4	–	**3**
PPy/MnO_2 composite	240 mF cm^{-2} (5 mV s^{-1})	1.0 M Na_2SO_4	62.3%	**4**
lCNT-GO/PPy	202.3 mF cm^{-2} (10 mV s^{-1})	1.0 M KCl	–	**5**
PPy/l-Ti_3C_2 film (l-Ti_3C_2, a MXene material)	203 mF cm^{-2}	0.5 M H_2SO_4	100% (20,000 cycles)	**6**
CNT/PANI hydrogel film	680 mF cm^{-2} (1 mA cm^{-2})	1 M H_2SO_4	–	**7**
CNT/PPy electrode	0.28 F cm^{-2} (1.4 mA cm^{-2})	0.05 M Na_2SO_4	–	**8**
RGO/PPy CCFs paper	363 mF cm^{-2} (0.5 mA cm^{-2})	PVA/H_3PO_4	–	**9**
Reduced graphene oxide/polypyrrole/cellulose hybrid papers	1.20 F cm^{-2} (2 mA cm^{-2})	1 M NaCl	89.5% (5000 cycles)	**10**
rGO/PPy films	411 mF cm^{-2} (0.2 mA cm^{-2})	1 M KCl	80% (5000 cycles)	**11**
Graphite/polyaniline hybrid electrodes on printing paper	355.6 mF cm^{-2} (0.5 mA cm^{-2})	1 M H_2SO_4	–	**12**
PPy-coated cotton fabrics	1325 mF cm^{-2} at 2 mA cm^{-2}	1 M Na_2SO_4	87% capacitance	**19**

			retention after 1000 charge/discharge cycles	
Polypyrrole/reduced graphene oxide coated fabric electrodes (Py–RGO-fabric)	265 F g^{-1} (5 mV s^{-1})	2.0 M NaCl	64% capacitance retention after 500 cycles	**20**
PANI/Au/paper electrode	800 mF cm^{-2} (1 mA cm^{-2})	1 M H_2SO_4	-	**21**

TABLE S2 Capacitive performances of all-solid-state supercapacitors reported in the literature and in this study

Electrode material	Specific capacitance of supercapacitor	Energy density	Power density	Ref.
**KF@PPy/*f*-CNT11**	**258 mF cm^{-2} (5 mV s^{-1})**<br>**219.4 mF cm^{-2} (0.5 mA cm^{-2})**<br>**3.44 F cm^{-3} (5 mV s^{-1})**	**22.3 μW h cm^{-2}**<br>**297.3 μW h cm^{-3}**	**2.1 mW cm^{-2}**<br>**28 mW cm^{-3}**	**This work**
lCNT-GO/PPy	70.0 mF cm^{-2} (10 mV s^{-1})	6.3 μW h cm^{-2}	3.7 mW cm^{-2}	5
CNT/PANI hydrogel film	184.6 F cm^{-2} (1 mA cm^{-2})	–	–	7
RGO/PPy CCFs paper	–	0.28 mW h cm^{-3}	20.9 mW cm^{-3}	9
rGO/PPy films	222 mF cm^{-2}	20 μW h cm^{-2}	5 mW cm^{-2}	11
Graphite/polyaniline hybrid electrodes on printing paper	77.8 mF cm^{-2} (0.1 mA cm^{-2})	0.32 mW h cm^{-3}	0.054 W cm^{-3}	12
CNT/PPy	4.9 F cm^{-3}	0.26 mW h cm^{-3}	0.15 W cm^{-3}	8
PPy/CNT/cotton fabric	50.9 mF cm^{-2} (10 mV s^{-1})	64.64 W h kg^{-1}	5.14 kW kg^{-1}	13
3D-graphene/graphite-paper	11 mF cm^{-2}	1.24 μW h cm^{-2}	1 mW cm^{-2}	14
PEDOT-GO/U–C electrode	30 mF cm^{-2} (10 mV s^{-1})	0.0022 mW h cm^{-2}	0.2 mW cm^{-2}	15
PANI-ZIF-67-CC	35 mF cm^{-2}	0.0161 mW h cm^{-3}	0.833 W cm^{-3}	16
PEDOT/H-15G-CNTF	37.8 mF cm^{-2} (5 mV s^{-1})	0.051 mW h cm^{-3}	2.1 mW cm^{-3}	17
MoS2@CNT/RGO electrode	29.7 mF cm^{-2} (0.1 mA cm^{-2})	4.13 μW h cm^{-2}	3.2 mW cm^{-2}	18

References

1. C. Yang, L. Zhang, N. Hu, Z. Yang, H. Wei and Y. Zhang, *Journal of Power Sources*, 2016, **302**, 39–45.
2. K. Shu, C. Wang, C. Zhao, Y. Ge and G. G. Wallace, *Electrochimica Acta*, 2016, **212**, 561–571.
3. C. Yang, L. Zhang, N. Hu, Z. Yang, H. Wei, Y. Wang and Y. Zhang, *Applied Surface Science*, 2016, **387**, 666–673.
4. X. Fan, X. Wang, G. Li, A. Yu and Z. Chen, *Journal of Power Sources*, 2016, **326**, 357–364.
5. H. Zhou and H. J. Zhai, *Organic Electronics*, 2016, **37**, 197–206.
6. M. Zhu, Y. Huang, Q. Deng, J. Zhou, Z. Pei, Q. Xue, Y. Huang, Z. Wang, H. Li, Q. Huang and C. Zhi, *Advanced Energy Materials*, 2016, **6,** 1600969.
7. S. Zeng, H. Chen, F. Cai, Y. Kang, M. Chen and Q. Li, *Journal of Materials Chemistry A*, 2015, **3**, 23864–23870.
8. Y. Chen, L. Du, P. Yang, P. Sun, X. Yu and W. Mai, *Journal of Power Sources*, 2015, **287**, 68–74.
9. S. Lyu, H. Chang, F. Fu, L. Hu, J. Huang and S. Wang, *Journal of Power Sources*, 2016, **327**, 438–446.
10. C. Wan, Y. Jiao and J. Li, *Journal of Materials Chemistry A*, 2017, **5**, 3819–3831.
11. J. Chen, Y. Wang, J. Cao, Y. Liu, Y. Zhou, J. H. Ouyang and D. Jia, *ACS Applied Materials & Interfaces*, 2017, **9**, 19831–19842.
12. B. Yao, L. Yuan, X. Xiao, J. Zhang, Y. Qi, J. Zhou, J. Zhou, B. Hu and W. Chen, *Nano Energy*, 2013, **2**, 1071–1078.
13. S. Huang, P. Chen, W. Lin, S. Lyu, G. Chen, X. Yin and W. Chen, *RSC Advances*, 2016, **6**, 13359–13364.
14. A. Ramadoss, K. Y. Yoon, M. J. Kwak, S. I. Kim, S. T. Ryu and J. H. Jang, *Journal of Power Sources*, 2017, **337**, 159–165.
15. D. Fu, H. Zhou, X.-M. Zhang, G. Han, Y. Chang and H. Li, *ChemistrySelect*, 2016, **1**, 285–289.
16. L. Wang, X. Feng, L. Ren, Q. Piao, J. Zhong, Y. Wang, H. Li, Y. Chen and B. Wang, *Journal of the American Chemical Society*, 2015, **137**, 4920–4923.
17. D. Fu, H. Li, X.-M. Zhang, G. Han, H. Zhou and Y. Chang, *Materials Chemistry and Physics*, 2016, **179**, 166–173.
18. S. Wang, J. Zhu, Y. Shao, W. Li, Y. Wu, L. Zhang and X. Hao, *Chemistry – A European Journal*, 2017, **23**, 3438–3446.

19. Y. Liang, W. Weng, J. Yang, L. Liu, Y. Zhang, L. Yang, X. Luo, Y. Cheng and M. Zhu, *RSC Advances*, 2017, **7**, 48934-48941.

20. J. Xu, D. Wang, Y. Yuan, W. Wei, L. Duan, L. Wang, H. Bao and W. Xu, *Organic Electronics*, 2015, **24**, 153-159.

21. L. Yuan, X. Xiao, T. Ding, J. Zhong, X. Zhang, Y. Shen, B. Hu, Y. Huang, J. Zhou and Z. L. Wang, *Angewandte Chemie International Edition*, 2012, **51**, 4934-4938.
